# Reviewed article: Jalil HF, Sartori LRM, Kohn HB, Gomes ECL, Xavier CB. Orofacial injuries in pregnant women victims of domestic violence:a crosssectional study at a forensic medicine service in Southern Brazil, 2015-2023. Epidemiol Serv Saude. 2025:34;e20240772

**DOI:** 10.1590/S2237-96222025v34e20240772.a

**Published:** 2025-10-27

**Authors:** Cristiane Colonese

**Affiliations:** 1 Reading Borough Council, Reading, UK, England Reading Borough Council Reading UK England

## Peer review A

## Questionnaire

Does the manuscript contain new and significant information to justify publication? Yes

Does the Abstract (Summary) clearly and accurately describe the content of the article? Yes

Is the problem significant and concisely stated? Yes

Are the methods described comprehensively? Yes

Are the interpretations and conclusions justified by the results? Yes

Is adequate reference made to other work in the field? Yes

## Manuscript Structure

Length of article is: Adequate

Number of tables is: Adequate

Number of figures is: Adequate

Please state any conflict(s) of interest that you have in relation to the review of this paper. None

Interest: Excellent

Quality: Good

Originality: Good

Overall: Good

Recommendation: Minor Revision

## Comments

Prezados autores, o artigo encontra-se bem escrito. Sugiro pequenas alterações. Página 1 - linha 15: rever escrita da frase. Página 3 - Sugiro que os autores usem apenas uma casa decimal ao descrever os dados da tabela. Não há a necessidade de informar que os dados são da tabela 1 ou 2 toda vez que descrever uma informação. Esse fato pode ser dito no início do parágrafo ou ao final. Sugiro que não sejam descritos dados estatísticos na discussão. Sugiro que seja revisto o uso da palavra AINDA como conector ao longo de todo o artigo. Os autores utilizam essa palavra de forma repetitiva. Na discussão seria interessante que os autores trouxessem outras pesquisas realizadas a nível nacional ou internacional sobre esse tema.

